# Effect of a synbiotic on functional abdominal pain in childhood

**DOI:** 10.22088/cjim.12.2.194

**Published:** 2021-03

**Authors:** Azade Gholizadeh, Sanaz Mehrabani, Mohammadreza Esmaeili Dooki, Mahmood Haji Ahmadi

**Affiliations:** 1 Student Research Committee, Babol University of Medical Sciences, Babol, Iran; 2 Non-Communicable Pediatric Diseases Research Center, Health Research Institute, Babol University of Medical Sciences, Babol, Iran

**Keywords:** Abdominal pain, Child, Synbiotic

## Abstract

**Background:**

One of the common functional gastrointestinal disorders in children is functional abdominal pain (FAP). The aim of the present study was to determine whether the administration of a synbiotic composed of fructo-oligosaccharides (FOS) and the seven types of beneficial bacteria is useful in FAP of childhood.

**Methods:**

In this placebo-controlled, double-blind trial, 4-15-year-old children who met the Rome III criteria for FAP were randomly divided to receive either synbiotic or placebo twice daily for 4 weeks. Primary outcome was at least 50% reduction in the number of pain episodes, and secondary outcomes were a decline of at least two scales in the pain duration and intensity based on Wong-Baker scale. Response to therapy was decrease of pain frequency/intensity.

**Results:**

A total of 67 children completed the trial (35 with synbiotic). Response rate was higher with synbiotic than placebo after four weeks (53.1 vs 11.4%; p<0.001), and synbiotic had significant superiority to placebo to relieve the duration (4.56±9.12 vs12±18.59, min/day, P=0.04), frequency (0.31±0.53vs 1.17±0.7, episode/Wk., P<.001) and intensity (2.38±2.29 vs 5.49±1.83, p<0.001) of abdominal pain.

**Conclusion:**

Synbiotic compared to placebo significantly decreased the intensity, frequency and duration of FAP in children.

Functional abdominal pain disorders (FAPDs) are common in children which can be divided into irritable bowel syndrome (IBS), abdominal migraine, functional abdominal pain (FAP) and functional dyspepsia (FD) (1, 2). The FAP is more common in schoolchildren with the prevalence of 8 and 10-15% in western countries (3, 4). According to the studies, the prevalence of recurrent abdominal pain was 11.8, 8 and 17% in 2-6-year-old children, middle school children and adolescents, respectively (5, 6). Among the children, the FAP has short- and long-term complications including lifetime psychiatric disorders, depression, social phobia, anxiety and somatic complaints (7). Although the patients’ burden is the poor quality of life as long-term consequences compared to the general population and patients with other chronic conditions (8). Absenteeism of school is an undesirable outcome in adolescents leading to school dropout, substance abuse and violence (7). There are no structural and biochemical abnormalities, except clinical symptoms to diagnose the FAP (9). Because the pathophysiology of FAP is not yet clear, there is a therapeutic challenge for pediatric gastroenterologists in children with FAP (4).

Now, some growing studies showed efficacy of probiotics as live micro-organisms with health benefits on the host in the treatment of different gastrointestinal disorders (10). But reports on synbiotics are few. Synbiotics are a combination of prebiotics (non-digestible foods which have beneficial effect on the host by stimulating the bacteria’s growth selectively) and probiotics. Due to the controversy over the use of probiotics in the treatment of FAP and lack of studies on the efficacy of synbiotics in pediatric patients, this placebo-controlled randomized trial was performed to evaluate the effects of synbiotic containing fructo-oligosaccharides and seven types of beneficial bacteria in the treatment of children who suffered from FAP.

## Methods

**Study design and participants: ** This double-blind, randomized, placebo-controlled clinical trial was conducted during one-year period (from December 2018 to December 2019) in a Pediatric Gastroenterology Clinic at Amirkola Children^’^s Hospital. Eligible participants were 4-14-year-old children who met the criteria of the Rome III diagnostic (continuous/episodic abdominal pain at least once a week during two months) for FAP, table 1 (11). The exclusion criteria were children with recent history (during the last month) or current treatment of antibiotics, antidepressants, antispasmodics, probiotics and prebiotics during 7 days prior to the study as well as children who had chronic underlying disease, known immune deficiency syndrome, alarm signs and symptoms including pain that causes the child to wake up from asleep, persistent right upper quadrant (RUQ) or right lower quadrant (RLQ) pain, severe vomiting (biliary, recurrent, periodic, or worrisome for a physician), dysphagia, unexplained fever, genitourinary symptoms, severe chronic or nocturnal diarrhea, unwanted weight loss, gastrointestinal bleeding, descending trend in growth curve, delayed puberty, localized tenderness in RUQ or RLQ, localized fullness or mass, splenomegaly, hepatomegaly, arthritis, jaundice, vertebral tenderness, costovertebral angle tenderness, perianal area disease, unexplained or abnormal findings in examination, hematochezia and anemia, positive family history of peptic and celiac ulcer disease, inflammatory bowel disease as well as chronic constipation.

To diagnose the FAP, all children were completely examined by a pediatric gastroenterologist. Moreover, the demographic data and frequency/intensity of abdominal pain were recorded by a single trained interviewer for whom the allocation sequence was blinded. The severity of pain was assessed via the Wong-Baker FACES® Pain Rating Scale containing six faces, indicating the effect of the pain. The ranges of this scale are from a relaxed face on the left (score 0 for no hurt) to a face, indicating that on the right, there is an intense pain (score 10 for worse hurts). The child was wanted to select at the time of pain which face she or he has (1), then the score of that face was considered for him/her. The pain frequency was determined with the number of pain episodes per week, the duration of abdominal pain was recorded per minute and the missing school days were considered for children >6 years old.

**Table 1:** The Rome III criteria for Childhood Functional Abdominal Pain&

The criteria are as follows:
1. Abdominal pain, continuously or episodically 2. Inadequate criteria for other FGIDs 3. No evidence of an anatomic, inflammatory, neoplastic and metabolic process which describes the symptoms of a patient
*Criteria achieved before diagnosis, at least once a week during 2 months

&. With permission from Rasquin A et al.

The Ethics Committee of Babol University of Medical Sciences approved the current study (IR.MUBABOL.HRI.REC.1397.270) and the Iranian Registry of Clinical Trials (IRCT:20190304042914N1) pre-registered it. The informed consent form was signed by parents of all selected children.

**Sample size and test power:** The sample size was calculated as 25 per group based on the predicted mean difference (∆=µ1- µ2, 7.2- 4.3=1.9) pain intensity between groups (12) with 5% significance level and 90% test power. Assuming estimated drop-out, 70 children were determined to be required.

**Intervention: **The FAP children were randomly categorized into synbiotic and placebo groups using a computer-generated randomization code. Both synbiotic and placebo sachets were manufactured by Zist Takhmir Pharmaceuticals Company (Tehran, Iran). Synbiotic sachets (Kidilact) composed of fructo-oligosaccharides (FOS) and seven types of beneficial bacteria including Lactobacillus casei, streptococcus thermophilus, Lactobacillus acidophilus, Lactobacillus bulgaricus, Lactobacillus rhamnosus, Bifidobacterium breve and Bifidobacterium infantis. The synbiotic group received synbiotic sachets twice daily for four consecutive weeks. The synbiotic and placebo boxes were the same shape; the placebo’s dimension, taste, appearance and indication were similar to those of the synbiotics, which represented that the ongoing study was blinded for patients and investigators. The placebo group received placebo sachets in a same order. Children did not use any prebiotics or probiotics other than those provided as well as the children and their parents were recommended to have a proper diet and avoid eating foods containing preservatives, carbonated drinks and fast food.

**Outcome measures and follow-up: **The primary outcome was at least 50% reduction in the number of pain episodes and secondary ones were a) a decline of at least two scales in the pain intensity based on face scaling, (2) a decrease of at least 50% in the pain duration, and (3) a decrease of at least 50% in the missing school days. Decrease of pain frequency/intensity was considered as response to treatment. 

If the children experienced any adverse events throughout the present study, the parents should visit or call the therapist. Adverse events were assessed during the second week of intervention by telephone interview and through a checklist including side effects of probiotics (bloating, diarrhea, itching and skin rash) at the end of the study. In cases with severe side effects, the medication was discontinued.

**Statistical analysis: **The SPSS 16 (Inc., Chicago, IL, USA) was applied to analyze the data. The outcomes of the study were compared based on per protocol. Data were shown as number (percentage) or mean±SD. Before analysis, the Kolmogorov Smirnov test was used for a normal distribution. Between-group comparisons were conducted via chi-square test and independent t-test. In addition, equivalent non-parametric tests were used if necessary. Statistically, in all analyses, a two-sided p<0.05 was set as a significant level.

## Results

**Figure 1:**
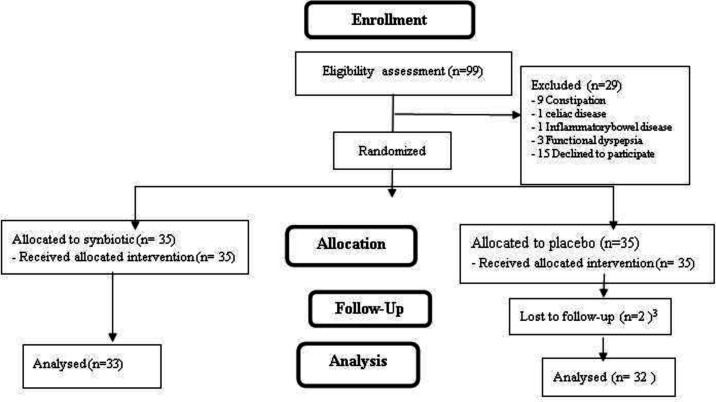
Diagram of consorting

Overall, the data were analyzed for 67 children (32 in the synbiotic group and 35 in the placebo group). Figure 1 illustrates the number of individuals who participated in the study for assessment through follow-up. Mean age and weight of the total participants were 7.61±2.69 years and 26.69±10.97 Kg, 45 (67.16%) of them were females as well as 29 (43.28%) cases were <7 years old, respectively. In terms of the baseline characteristics, no significant differences were found between the subjects (table 2).

**Table 2:** Comparison of demographic data and baseline characteristics between two groups

	**Synbiotic**	**Placebo**	**P.value**
Age, mean± SD*, y*	7.32±2.57	7.88±2.81	0.42
Male/female, n*/n	9.23	13.22	0.43
Weight, Kg	25.26±9.18	28±12.38	0.38
Frequency of pain, means±SD,n/wk*	1.06±.1.10	1.40±0.97	0.17
Pain intensity, mean±SD	6.19±1.55	6.11±1.81	0.93
School absenteeism because of pain, n	10	8	0.15
Pain duration, means±SD, Min*/day	34.16±27.41	33.03±27.41	0.90
Age group ≤7years > 7 years	15 (46.9%) 17 (53.1%)	14 (40%) 21 (60%)	0.28

N: number, Min:minute,Wk:week, Y:year, SD:standard deviation.

**Primary and secondary outcome measures: **The comparisons between two groups in terms of the primary and secondary outcomes are presented in table 3. Episode less than one time per week was observed in 15 children; 14(93.3%) children were in the synbiotic group (p<0.001). Out of 38 children who had pain duration <5 minute per episode, 22(57.9%) ones were in the synbiotic group (P=0.007). At the end of the follow-up, the response to treatment was observed in 17 children in the synbiotic group and 4 children in the placebo group (53.1% vs 11.4%; p<0.001).

**Table 3:** Comparison of primary and secondary outcomes between two groups

	**Synbiotic**	**Placebo**	**P.value**
Frequency of pain, means±SD*, n*/week	0.31±0.53	1.17±0.7	<0.001
Intensity of pain, mean±SD	2.38±2.29	5.49±1.83	<0.001
Duration of pain, means±_SD, min*/day	4.56±9.12	12±18.59	0.040

*. Min: minute, SD: standard deviation, N: number.

The comparison of within groups was significant in synbiotic group in terms of pain frequency, severity and duration (p<0.001) as well was significant in pain severity (P=0.009) and duration (p<0.001) in placebo group. There were no adverse events and absenteeism of school in both groups during the study period.

## Discussion

This prospective randomized study evaluated the patients’ response when there was a decrease in severity, duration and frequency of pain. In addition, the present results suggested that the synbiotics versus placebo could provide better outcome to control the pain in FAP children. At the end of the fourth week, neither adverse reactions nor side effects were observed in both groups.  The therapeutic effect of synbiotics is not yet clear. It is possible that synbiotics act through stimulating the probiotics by prebiotics, leading to the metabolic activity’s modulation in the intestine with the inhibition of potential pathogens, existent in the gastrointestinal tract, development of beneficial microbiota and maintenance of the intestinal biostructure (13).

There are many studies on the effect of probiotics in functional gastrointestinal disorder (FGID), but there are few placebo-controlled trials of therapeutic effects of synbiotics on gastrointestinal disorders, for example, pain-related FGIDs and IBS, especially in pediatric patients. Several studies suggested that the synbiotic supplements were effective on weight loss and some cardio-metabolic risk factors among children and adolescents (14-16). Saneian et al. (4) used synbiotics containing Bacillus coagulants (LGG) and FOS in 88 children suffered from FAP according to the Rome III criteria in a double-blind controlled randomized trial, and the response rate was 60% in the synbiotic and 39.5% in placebo groups after 4 weeks, but no significant difference was found in 12-week follow-up. 

Besides, there was no difference between two groups in terms of pain severity in the 4th and 12th weeks. Other study compared the effects of synbiotics (LGG + FOS), placebo and peppermint oil on abdominal pain-related FGID and demonstrated that the peppermint oil was superior to synbiotics in reducing duration and severity of pain. However, the mentioned study used peppermint oil only for IBS patients (17). A meta-analysis study indicated that the LGG was effective in treatment of pain-related FGIDs, particularly IBS in children, and no therapeutic effects were found for children with FAP or functional dyspepsia (18). These findings are in agreement with those of Francavilla et al. (3). Weizman et al. (12) and Romano et al. (19) found that the L. reuteri DSM 17938 versus placebo decreased significantly the intensity and frequency of FAP in children aged 6-15 years during the 12-week follow-up. A systematic review by Ding et al. (20) displayed that the utilization of probiotic Lactobacillus rhamnosus GG did not have significant effect on pain relief in children with FAP, while probiotic Lactobacillus reuteri was effective in reducing the severity of pain in FAP children. The current study is important in two respects: First, the use of synbiotics is a proper combination of both prebiotics and probiotics in a single product along with a superior activity in comparison with the effect and role of the probiotic or prebiotic alone (21), and second, the synbiotics have seven types of beneficial bacteria such as Bifidobacterium infantis, Lactobacillus casei, Bifidobacterium breve, Lactobacillus bulgaricus, streptococcus thermophiles, Lactobacillus rhamnosus and Lactobacillus acidophilus. Furthermore, various types of probiotic bacteria had different effects on the basis of enzymatic activities and specific capabilities, even within one species (12); hence, these conflicting results can explain the differences in findings of the studies with similar and even different probiotics as well as can warrant further studies. It is recommended to perform more studies with larger sample size and longer follow-up as well as determine the micro-bits to select the most appropriate probiotic combination in the treatment of children with FAP. Of course, according to the Rome committee recommendation, the follow-up for at least 6 months is necessary to create a treatment efficacy for FGIDs during a long time.

There were some limitations in the present study. First, using a self-reported face pain scale was a subjective tool to detect the intensity of abdominal pain. Nevertheless, it was a valid scale in children and was used commonly in other studies. Second, the comparison of within placebo group was unexpectedly significant, and it seemed that a larger sample size was required. Another limitation was the lack of long-term follow-up of patients to better evaluate the beneficial effects of synbiotics in the treatment of FAP. Finally, there was no study which used the same synbiotics; thus, it is impossible to compare the results more accurately.

In conclusion the used synbiotics significantly reduced the severity, frequency and duration of abdominal pain in FAP children during a 4-week trial without side effects.
